# Supplementary material for: Identifying Current Feelings of Mild and Moderate to High Depression in Young, Healthy Individuals Using Gait and Balance: An Exploratory Study
Source: Sensors (Basel). 2023 Jul 23;23(14):6624. doi: 10.3390/s23146624 (PMC10384769; doi:10.3390/s23146624)
Supplement: Supplementary file 1 [file sensors-23-06624-s001.zip › Screening_Questionnaire.pdf]

Appendix F: Screening Survey

ID______________ Date: _________________

Please answer YES or NO to the following questions:

1. Do you have any limitations to perform physical activity such as walking? _____
2. Do you experience pain/discomfort when walking or standing from a sitting position? _____
3. Have you ever had a stroke? _____
4. Have you ever been diagnosed with a neurological condition? _____
5. Have you had an orthopedic surgery within the last six months? _____
6. Do you currently have a wound on your feet? _____
7. Have you noticed any decrease or change in sensation on the bottom of your feet? _____
8. Do you have difficulty seeing full range of colors? _____
9. Have you ever been diagnosed with color blindness? _____
10. Have you ever been diagnosed with a mental health condition? _____
11. Do you wear glasses? _____

    *If you answer yes, please wear your glasses/contact lenses when participating in this study
