# Supplementary material for: Identifying Current Feelings of Mild and Moderate to High Depression in Young, Healthy Individuals Using Gait and Balance: An Exploratory Study
Source: Sensors (Basel). 2023 Jul 23;23(14):6624. doi: 10.3390/s23146624 (PMC10384769; doi:10.3390/s23146624)
Supplement: Supplementary file 1 [file sensors-23-06624-s001.zip › SupplementalTable.pdf]

Supplemental Table S1-Quality Assessment Tool for Before-After (Pre-Post) Studies With No Control Group

Criteria	Yes<br>Location	No<br>Comments	Other (CD, NR, NA)<br>Comments
1. Was the research question or objective in this paper clearly stated?	☑<br>Introduction		
2. Was the study population clearly specified and defined?	☑<br>Participants<br>Results		
3. Was the participation rate of eligible persons at least 50%?			☒<br>Exploratory, hypothesis-generating; no extrapolation was aimed
4. Were all eligible participants that met the prespecified entry criteria enrolled?	☑<br>Participants		
5. Was the sample size sufficiently large to provide confidence in the findings?		☒<br>Exploratory; no prespecified question	
6. For the analyses in this paper, were the exposure(s) of interest measured prior to the outcome(s) being measured?		☒<br>Cross-sectional	
7. Were the outcome measures prespecified, clearly defined, valid, reliable, and assessed consistently across all study participants?		☒<br>Cross-sectional	
8. For exposures that can vary in amount or level, did the study examine different levels of the exposure as related to the outcome (e.g., categories of exposure, or exposure measured as continuous variable)?		☒<br>Preferred levels of outcome variable	
9. Were the exposure measures (independent variables) clearly defined, valid, reliable, and implemented consistently across all study participants?	☑<br>Instruments		
10. Was the exposure(s) assessed more than once over time?			N/A<br>Cross-sectional
11. Were the outcome measures (dependent variables) clearly defined, valid, reliable, and implemented consistently across all study participants?	☑<br>Instruments		
12. Were the outcome assessors blinded to the exposure status of participants?	☑<br>Statistical Analyses		

13. Was loss to follow-up after baseline 20% or less?			N/A Cross-sectional
14. Were key potential confounding variables measured and adjusted statistically for their impact on the relationship between exposure(s) and outcome(s)?	☑ Statistical Analyses		

Note. In order to make this table more user-friendly, we added the Location for when we satisfied a criterion and the Comments for when we did not; CD = cannot determine; NA = not applicable; NR = not reported
